# Clinical effectiveness of fresh frozen plasma compared with fibrinogen concentrate: a systematic review

**DOI:** 10.1186/cc10488

**Published:** 2011-10-14

**Authors:** Sibylle Kozek-Langenecker, Benny Sørensen, John R Hess, Donat R Spahn

**Affiliations:** 1 Department of Anaesthesia and Intensive Care, Evangelical Hospital Vienna, Hans-Sachs-Gasse 10-12, 1180-Vienna, Austria; 2 Haemostasis Research Unit, Centre for Haemostasis and Thrombosis, Guy's and St Thomas' Hospital & King's College London School of Medicine, Westminster Bridge Road, London, SE1 7EH, UK; 3 Centre for Haemophilia and Thrombosis, Aarhus University Hospital, Skejby-Brendstrupgårdsvej 100, Skejby, 8200, Denmark; 4 Department of Pathology, University of Maryland School of Medicine, 10 South Pine Street, MSTF, Baltimore, MD 21201-1192, USA; 5 Institute of Anaesthesiology, University Hospital Zurich, Raemistrasse 100, CH-8091 Zurich, Switzerland

## Abstract

**Introduction:**

Haemostatic therapy in surgical and/or massive trauma patients typically involves transfusion of fresh frozen plasma (FFP). Purified human fibrinogen concentrate may offer an alternative to FFP in some instances. In this systematic review, we investigated the current evidence for the use of FFP and fibrinogen concentrate in the perioperative or massive trauma setting.

**Methods:**

Studies reporting the outcome (blood loss, transfusion requirement, length of stay, survival and plasma fibrinogen level) of FFP or fibrinogen concentrate administration to patients in a perioperative or massive trauma setting were identified in electronic databases (1995 to 2010). Studies were included regardless of type, patient age, sample size or duration of patient follow-up. Studies of patients with congenital clotting factor deficiencies or other haematological disorders were excluded. Studies were assessed for eligibility, and data were extracted and tabulated.

**Results:**

Ninety-one eligible studies (70 FFP and 21 fibrinogen concentrate) reported outcomes of interest. Few were high-quality prospective studies. Evidence for the efficacy of FFP was inconsistent across all assessed outcomes. Overall, FFP showed a positive effect for 28% of outcomes and a negative effect for 22% of outcomes. There was limited evidence that FFP reduced mortality: 50% of outcomes associated FFP with reduced mortality (typically trauma and/or massive bleeding), and 20% were associated with increased mortality (typically surgical and/or nonmassive bleeding). Five studies reported the outcome of fibrinogen concentrate versus a comparator. The evidence was consistently positive (70% of all outcomes), with no negative effects reported (0% of all outcomes). Fibrinogen concentrate was compared directly with FFP in three high-quality studies and was found to be superior for > 50% of outcomes in terms of reducing blood loss, allogeneic transfusion requirements, length of intensive care unit and hospital stay and increasing plasma fibrinogen levels. We found no fibrinogen concentrate comparator studies in patients with haemorrhage due to massive trauma, although efficacy across all assessed outcomes was reported in a number of noncomparator trauma studies.

**Conclusions:**

The weight of evidence does not appear to support the clinical effectiveness of FFP for surgical and/or massive trauma patients and suggests it can be detrimental. Perioperatively, fibrinogen concentrate was generally associated with improved outcome measures, although more high-quality, prospective studies are required before any definitive conclusions can be drawn.

## Introduction

Surgery and massive trauma are frequently associated with significant derangement of haemostatic capacity caused by loss, consumption, endogenous inhibition (by the protein C pathway), dilution of coagulation factors (termed 'dilutional coagulopathy' or 'acute coagulopathy of shock and trauma') and increased clot breakdown (fibrinolysis). Most commonly, haemostatic therapy in patients without preexisting haemostatic disorders aims to substitute key components (clotting factors and other blood components) by transfusion of allogeneic blood products, including fresh frozen plasma (FFP), platelet concentrate (PC), packed red blood cells (RBC) and, in some countries, cryoprecipitate.

Clinical use of FFP in component therapy has increased over the past four decades [1]. Reports of the success of high FFP:RBC ratios may be responsible for some of this rise [2-4]. The benefits of any intervention should outweigh the risks. FFP has been associated with increased risk of morbidity and mortality [5,6], whilst the evidence to support the effectiveness of FFP in the perioperative setting or its appropriate dosing in the massive transfusion setting has been questioned [7-10].

Fibrinogen is a key protein in the new 'cell-based' model of haemostasis that involves four consecutive overlapping stages (initiation, amplification, propagation and stabilisation), in which the conversion of fibrinogen to a covalently linked fibrin network is the final stage [11,12]. Of note, fibrinogen is the first coagulation factor to fall to a suboptimal level early during bleeding and dilutional coagulopathy [13]. Fibrinogen supplementation is therefore recommended in patients with massive bleeding to maintain plasma fibrinogen levels above 1.5 to 2.0 g/L [14]. Supplementation of fibrinogen may be most effective when performed as part of early goal-directed coagulation management [15]. Typically, standard preparation FFP contains 2.0 g/L (range = 0.9 to 3.2 g/L) fibrinogen (equivalent to 0.6 g in a 300-ml unit), as well as other pro- and anticoagulant factors found in plasma, acute phase proteins (cytokines), electrolytes, immunoglobulins and albumin [9,16]. Fibrinogen concentrate (from various manufacturers) is currently licensed for use in congenital bleeding in China, Japan, the USA and throughout Europe, and for use in acquired bleeding in > 15 countries globally. Pasteurised and lyophilised human fibrinogen concentrate is typically reconstituted in 50 ml of sterile water to a final concentration of 20 g/L. Each vial of fibrinogen concentrate contains 1.0 g (range = 0.9 to 1.3 g) of fibrinogen, as well as albumin, L-arginine hydrochloride, sodium chloride and sodium citrate [17]. The concentrated dose of fibrinogen provided by fibrinogen concentrate (final concentration 10 times higher than FFP) might be preferable to FFP for restoring plasma fibrinogen levels because of its rapid availability (no thawing), reduced volume (faster infusion time) and increased safety, and it has recently been suggested to be more effective than administration of FFP [15]. However, despite growing evidence based on both preclinical and clinical studies to support its efficacy and safety [17-20], fibrinogen concentrate is not yet a standard component of many transfusion protocols.

This systematic review was performed to investigate the evidence base for the use of FFP and fibrinogen concentrate in the perioperative and/or massive trauma setting. We have included observational studies and case series in addition to randomised, controlled trials (RCTs) to provide a comprehensive overview of the current clinical situation.

## Materials and methods

### Study concept and administrative structure

The initial rationale behind this systematic review was formulated by the lead author (SKL), who subsequently recruited the coauthors, arranged for an unrestricted educational grant from CSL Behring (Marburg, Germany), a manufacturer of fibrinogen concentrate, and liaised closely with Fishawack Communications (Oxford, UK), which provided editorial support. Within this structure, we had full control of the ideas, data, reporting and conclusions of the research and were not influenced by any commercial activities. Our study did not require ethical approval.

### Search criteria

Studies reporting the administration of FFP or fibrinogen concentrate in a perioperative or massive trauma setting were identified in MEDLINE and EMBASE (1 January 1995 to 31 December 2010). The reference lists of identified studies and relevant reviews were checked for additional citations and to identify key FFP RCTs conducted prior to 1995.

In the electronic database search, we used 'perioperative', 'surgery', 'h(a)emostasis', 'bleeding', 'traumatic injuries', 'blood component transfusion' as both input words and subject headings. The FFP search also included 'fresh frozen plasma', 'FFP', 'plasma' and 'therapeutic plasma', and the fibrinogen concentrate search comprised 'fibrinogen/therapeutic use', 'fibrinogen concentrate', 'fibrinogen concentrates'. Additional limits were added: (1) language: English or German; (2) date range: 1 January 1995 to 31 December 2010; and (3) article type: clinical trial, meta-analysis, RCTrandomized controlled trial, case reports, clinical trial phases I to IV, comparative study, controlled clinical trial and journal article. Titles and abstracts were screened for relevance, and full publications of the potentially relevant studies were assessed against the formal eligibility criteria.

### Eligibility criteria

Studies were eligible for inclusion in the systematic review if they reported a predefined outcome of the administration of FFP or fibrinogen concentrate specifically within the perioperative or massive trauma setting. All preparations of FFP ('standard' as well as 'pathogen-reduced') were eligible for inclusion. Abstracts of work presented at congresses were excluded.

Outcomes of interest included blood loss, allogeneic transfusion requirements, survival, hospital and/or ICU length of stay (LOS), plasma fibrinogen levels, thrombotic events, acute lung injury (ALI), transfusion-associated circulatory overload (TACO), infections (bacterial contamination and viral transmission) and multiple organ failure (MOF). A meta-analysis of the effect of FFP on morbidity was recently published by Murad and colleagues [5]. To avoid redundancy, outcomes related to morbidity (thrombotic events, ALI, TACO, infections [bacterial contamination and viral transmission] and MOF) were not extracted and tabulated for FFP studies.

All study types were included (RCTs, non-RCTs, prospective comparator studies, retrospective comparator studies, prospective and retrospective noncomparator studies, and case reports). Study design and quality of evidence were captured at the data extraction stage.

Studies were included regardless of patient age, sample size or duration of patient follow-up. We excluded preclinical studies, studies which included patients with congenital clotting factor deficiencies or other haematological disorders and studies reporting the prophylactic administration of FFP or fibrinogen concentrate in the absence of surgery. Studies reporting mixed populations were included only if > 50% of the cases were surgical and/or trauma patients. Studies reporting the administration of FFP for liver disease or reversal of vitamin K anticoagulation were excluded if there was no surgical intervention. Studies in which control groups received autologous platelet-rich plasma were also excluded. For assessment of the effect of each intervention on survival, studies that reported no deaths in either study arm were excluded from the survival outcome but included in all other applicable outcomes of interest.

### Data extraction

Data from eligible studies were extracted according to the population, intervention, comparison and outcome (PICO) method into a standard form and included study size, study type, number of study arms (including the presence of a control group), the number and age (paediatric or adult) of patients in each arm, indication for and nature of the intervention (including the control group where applicable), the presence of chronic liver disease, treatment with anticoagulants, methods for measuring (where applicable) blood loss and fibrinogen levels, and relevant outcome data. Where possible, blood loss and transfusion requirements were recorded as intra-, peri- or postoperative (where perioperative was the intra- and postoperative periods combined), and survival rates were recorded by the time period reported (6 hours, 24 hours, 30 days, in-hospital, and so on). Consequently, in some instances, multiple outcomes from a single study were included.

### Analysis

Studies were categorised by type, then assessed by the effect of the intervention on each applicable outcome criterion. Each intervention (FFP or fibrinogen concentrate) could show a positive, negative or null effect against the comparator group or against absolute values where no comparator group was available. As such, data from the observational and case studies were generally descriptive in nature and were only included as supportive evidence. Studies without comparator groups were not included in any formal calculations of the efficacy of the intervention. Where outcome measures were compared between groups within a study, these comparisons were assessed on the basis of a difference in group averages (mean or median as available). The primary purpose of this review was achieved by describing the numbers and the quality of studies identified in relation to the effect of FFP or fibrinogen concentrate on the stated outcome measures. To avoid redundancy and the inclusion of multiple instances of data from the same study, meta-analyses were not intended to be formally included in the analysis (that is, outcomes from meta-analyses were not counted towards the number of studies reporting the effect of an intervention for each outcome). Instead, meta-analyses were included descriptively as supportive evidence. Meta-analysis was not a stated aim of this review, and analysis of the data extracted from the eligible trials formed the basis of the conclusions reached.

## Results

### Studies included

**Figure 1:**
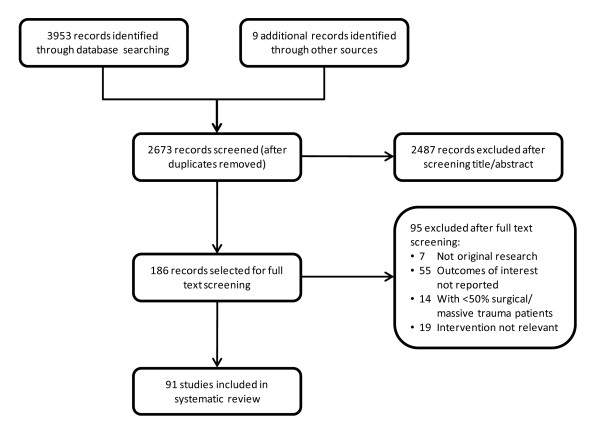
**Study flowchart**.

The selection process and study flow are depicted in Figure 1. The majority of identified citations were easily excluded at the level of title or abstract on the basis of no relevance to this review. After screening, there were 91 studies that fulfilled our eligibility criteria, reported outcomes of interest and were formally included in the analysis (Tables 1, 2 and 3). There was also one meta-analysis that was used as supportive evidence. Only a minority of selected studies (*n *= 18) were high-quality prospective studies with randomisation procedures and a control arm (Table 4). A further 39 nonrandomised and observational studies with a comparator group were included. The majority (62%) of FFP studies involved the comparison of the intervention group against a different dosage (typically by assessing the effect of different FFP:RBC ratios), formulation (various kinds of 'pathogen-reduced' plasmas) or blood product (for example, cryoprecipitate or whole blood). There were only 20 studies in which FFP was compared with no FFP or with a non blood product (for example, colloid or crystalloid). There were five fibrinogen concentrate comparator studies, two comparing fibrinogen concentrate with no fibrinogen concentrate or with a non blood product and, importantly, three studies comparing the effect of fibrinogen concentrate directly with FFP (administered in combination with other allogeneic products).

**Table 1:** Summary details of eligible randomised, controlled trials

Study	Indication	Study type	*N *patients (intervention group vs controls)	Intervention details	Control group
FFP					
Boyd *et al.* (1996) [108]	Surgery (renal transplantation; renal disease and coagulopathy)	RCT	39 (19 vs 20)	FFP 2 U	Conjugated oestrogen 50 mg
Chong Sung *et al. *(2006) [35]	CV surgery (paediatric)	RCT	42 (21 vs 21)	FFP 10 ml/kg	HES (130/0.4) 10 ml/kg
Consten *et al.* (1996) [36]	CV surgery	RCT	50 (24 vs 26)	FFP 3 U	Plasma substitute 3 U
Demeyere *et al*. (2010) [37]	CV surgery in patients on OAT	RCT	40 (20 vs 18)	FFP 4 U (additional doses could be administered to reach target INR)	PCC calculated to target INR of 1.5 (additional doses could be administered to reach target INR)
Etemadrezaie *et al. *(2007) [78]	Traumatic brain injury	RCT	90 (44 vs 46)	FFP 10 to 15 ml/kg	Saline 10 to 15 ml/kg
Haubelt *et al.* (2002) [38]	CV surgery	Block-RCT	67 (31 vs 36)	FFP 600 ml	SD-FFP 600 ml
Kasper *et al.* (2001) [41]	CV surgery	RCT	56 (27 vs 29)	FFP 15 ml/kg	6% HES (450/0.7) 15 ml/kg
Laine *et al.* (2003) [64]	Liver transplantation	RCT	33 (15 vs 18)	FFP median 14 U (+10 U RBC)	WB median 12 U
Menges *et al.* (1992) [43]	Surgery (other)	RCT	42 (16 vs 12/14)	(Autologous) FFP + blood (dose not reported)	Homologous RBC/autologous blood (dose not reported)
Mintz *et al.* (2006) [65]	Liver transplantation	RCT	51 (29 vs 22)	FFP 22.7 U over 7 days	PCT-FFP 21.3 U over 7 days
Noddeland *et al*. (2002) [45]	CV surgery	RCT	84 (55 vs 29)	SD-FFP (2:1 Uniplas:Octaplas) median 3 U Uniplas:2 U Octaplas	No FFP
Trimble *et al.* (1964) [48]	CV surgery (adults, children)	RCT	53 (21 vs 32)	FFP 2 U adults, 1 U children	No FFP
von Sommoggy *et al. *(1990) [49]	Surgery (other)	RCT	24 (13 vs 11)	FFP (+ human albumin) mean 2.7 U	HES (dose not reported)
Wilhelmi *et al.* (2001) [50]	CV surgery	Block-RCT	120 (60 vs 60)	FFP 4 U (1,000 ml)	HES 1,000 ml
Williamson *et al.* (1999) [22] (duplicate data in [21])	Surgery (liver)	RCT	25 (13 vs 12)	FFP median 44 ml/kg	SD-FFP median 44 ml/kg
Fibrinogen concentrate					
Cui *et al.* (2010) [56]	CV surgery (children)	RCT	31 (17 vs 14)	Fibrinogen concentrate 0.5 to 1.0 g	'Standard allogeneic therapy'
Fenger- Eriksen *et al*. (2009) [71]	Surgery (cystectomy)	RCT	20 (10 vs 10)	Fibrinogen concentrate 45 mg/kg	Placebo (isotonic saline) 2.25 ml/kg
Karlsson *et al.* (2009) [54]	CV surgery	RCT	20 (10 vs 10)	Fibrinogen concentrate 2 g	No fibrinogen concentrate

CV = cardiovascular; FFP = fresh frozen plasma; HES = hydroxyethyl starch; INR = International Normalised Ratio; OAT: oral anticoagulant therapy; PCT-FFP: photochemical treatment fresh frozen plasma; PCC: prothrombin complex concentrate; RBC: packed red blood cells; RCT = randomised, controlled trial; SD-FFP = solvent/detergent-treated fresh frozen plasma; WB = whole blood.

**Table 2:** Summary details of eligible comparator trials

Study	Indication	Study type	*N *(intervention group vs controls)	Intervention details	Control group
FFP					
Antolovic *et al.* (2008) [75]	Surgery (ischaemic colitis)	Retrospective comparator	81 (30 vs 51)	FFP ≥ 3 U	FFP < 3 U
Apaydin *et al.* (2002) [76]	CV surgery	Retrospective comparator	85 (16 vs 69)	FFP > 2 U	FFP ≤ 2 U
Bochicchio *et al.* (2008) [77]	Trauma	Prospective comparator	922	FFP mean 5.4 U	No FFP
Borgman *et al.* (2007) [34]	Massive trauma	Retrospective comparator	246	FFP:RBC high 1:1.4	FFP:RBC 1:2.5 and 1:8
Chowdhury *et al.* (2004) [109]	Various	Prospective comparator	22 (10 vs 12)	FFP standard dose median 12.2 ml/kg	FFP high dose median 33.5 ml/kg
Dente *et al.* (2009) [30]	Massive trauma	Prospective cohort vs historical control	157 (73 vs 84)	FFP:RBC ≥ 1:2 (MTP)	FFP:RBC < 1:2 (pre MTP)
Duchesne *et al.* (2008/2009/2010) [31-33]	Massive trauma, surgery (TIC), trauma	Retrospective comparator	385/135/196	FFP:RBC 1:1	Ratios outside 1:1
Gunter (2008) *et al. *[28]	Massive trauma	Prospective cohort vs historical control	259 (64 vs 195)	FFP:RBC 1:1 to 1:1.5	FFP:RBC < 1:1.5 and > 1:1
Hildebrandt (2007) *et al. *[23] and Kerner *et al.* (2008) [24]	Surgery (craniofacial, infants)	Non RCT	30 (15 vs 15)	FFP mean 45 ml/kg	5% human albumin
Holcomb *et al.* (2008) [3]	Massive trauma	Retrospective comparator	466	FFP:RBC ≥ 1:2	FFP:RBC < 1:2
Inaba *et al. *(2010) [79]	Trauma (nonmassive transfusion)	Retrospective matched cohort	568 (284 vs 284)	FFP mean 3.0 U	No FFP
Johansson *et al.* (2007) [39]	CV surgery	Prospective cohort vs historical control	148 (55 vs 93)	FFP:RBC 1:1 (transfusion package) mean 10 U	FFP:RBC < 1:1 (pre transfusion package) mean 7 U
Johansson *et al.* (2009) [69]	Surgery (various) with massive transfusion	Prospective cohort vs historical control	832 (442 vs 390)	FFP:RBC 1:1.3 (transfusion package)	FFP:RBC 1:1.6 (pre transfusion package)
Kaibori *et al.* (2008) [40]	Surgery (hepatectomy for hepatocellular carcinoma)	Retrospective comparator	184 (43 vs 141)	FFP median 4.0 U	No FFP
Kashuk *et al.* (2008) [80]	Massive trauma	Retrospective comparator	133	FFP:RBC 1:1	FFP:RBC 1:2, 1:3, 1:4 or 1:5
Koch *et al. *(2009) [105]	CV surgery	Retrospective matched cohort	1928 (964 vs 964)	FFP, dose not reported	No FFP
Maegele *et al.* (2008) [81]	Massive trauma	Retrospective comparator	713	FFP:RBC 1:1	FFP:RBC < 0.9:1 and > 1.1:1
Massicotte *et al.* (2005) [82]	Liver transplantation	Retrospective comparator	66 (26 vs 40)	FFP mean 4.9 U	No FFP
Mell *et al. *(2010) [42]	CV surgery	Retrospective comparator	128 (87 vs 41)	FFP:RBC ≥ 1:2	FFP:RBC < 1:2
Miller *et al. *(1997) [44]	CV surgery (paediatric)	Prospective comparator	36 (19 vs 17)	FFP, dose not reported	Cryoprecipitate, dose not reported
Mitra *et al. *(2010) [66]	Massive trauma	Retrospective comparator	331	FFP:RBC > 1:1.5	FFP:RBC < 1:1.5
Oberkofler *et al.* (2010) [83]	Liver transplantation	Retrospective comparator	144	FFP > 10 U	FFP < 10 U
Ono *et al. *(2002) [84]	CV surgery	Retrospective comparator	60 (39 vs 21)	FFP 3.2 U	No FFP
Reed *et al. *(1986) [46]	Trauma	Prospective cohort vs published data	40 (16 vs 24)	FFP 2 U/12 U blood	No FFP
Riskin *et al.* (2009) [85]	Massive trauma	Retrospective comparator	77	FFP:RBC 1:1.8 (MTP)	FFP:RBC 1:1.8 (pre MTP)
Rose *et al. *(2009) [86]	Massive trauma	Retrospective comparator	204	FFP:RBC 1:1	FFP:RBC < 0.9:1 and > 1.1:1
Roy *et al. *(1988) [47]	CV surgery	Retrospective comparator (sequential cohorts)	100 (52 vs 48)	FFP mean 5.8 U	5% albumin
Scalea *et al. *(2008) [87]	Trauma	Prospective comparator	250	FFP:RBC 1:1	Ratios outside 1:1
Scheele *et al.* (2001) [88]	Surgery (liver resection for colorectal metastases)	Retrospective comparator	425 (159 vs 308)	FFP, dose not reported	No FFP
Shaz *et al. *(2010) [4]	Massive trauma	Retrospective comparator	214	FFP:RBC ≥ 1:2	FFP:RBC < 1:2
Snyder *et al.* (2009) [89]	Massive trauma	Retrospective comparator	134 (60 vs 74)	FFP:RBC ≥ 1:2	FFP:RBC < 1:2
Sperry *et al.* (2008) [67]	Massive trauma	Retrospective comparator	415	FFP:RBC ≥ 1:1.5	FFP:RBC < 1:1.5
Stinger *et al.* (2008) [90]	Massive trauma	Retrospective comparator	252	FFP high fibrinogen:RBC > 0.2 g fibrinogen/1 U RBC)	FFP low fibrinogen:RBC < 0.2 g fibrinogen/1 U RBC)
Teixeira *et al.* (2009) [91]	Massive trauma	Retrospective comparator	383	FFP:RBC > 1:2	FFP:RBC < 1:8, 1:8 to 1:3 and 1:3 to 1:2
Tomimaru *et al.* (2010) [92]	Surgery (hepatectomy for hepatocellular carcinoma)	Retrospective comparator	497 (204 vs 293)	FFP median 10 U	No FFP (in 93% of patients in the group)
Wright *et al.* (2008) [93]	CV surgery	Retrospective comparator	211 (129 vs 82)	FFP (mixed donors) median 6 U	FFP (male-only donors) median 4 U
Zink *et al. *(2009) [68]	Massive trauma	Retrospective comparator	466	FFP:RBC ≥ 1:1	FFP:RBC < 1:1
Fibrinogen concentrate					
Rahe-Meyer *et al.* (2009) [55]	CV surgery	Non RCT	15 (10 vs 5)	Fibrinogen concentrate mean 5.7 g	FFP mean 4.2 U
Rahe-Meyer *et al.* (2009) [26]	CV surgery	Prospective cohort vs historical control	18 (6 vs 12)	Fibrinogen concentrate mean 7.2 g	FFP mean 9.1 U

CV = cardiovascular; FFP = fresh frozen plasma; MTP = massive transfusion protocol; RBC = packed red blood cells; RCT = randomised, controlled trial; TIC = trauma-induced coagulopathy.

**Table 3:** Summary details of eligible noncomparator trials

Study	Indication	Study type	Patients (*N*)	Intervention details	Control group
FFP					
Aiyagari *et al. *(2005) [51]	Trauma (gunshot injury)	Case report	3	FFP 10 U, 4 U, 8 U	No control group
Dann *et al. *(2008) [110]	Massive trauma	Retrospective noncomparator	21	FFP:RBC 1:1.5	No control group
Díaz-Gómez *et al. *(2010) [94]	CV surgery	Retrospective noncomparator	14,868	FFP	No control group
Fan *et al. *(2003) [95]	Liver transplantation	Prospective noncomparator	100	FFP	No control group
Fischer *et al. *(2008) [52]	GI bleeding (preterms)	Retrospective noncomparator	5	FFP 10 to 30 ml/kg/30 minutes	No control group
Gonzalez *et al. *(2007) [96]	Massive trauma	Retrospective noncomparator	97	FFP mean 5 U	No control group
Lei *et al. *(2009) [106]	CV surgery	Retrospective noncomparator	298	FFP mean 1,090 ml	No control group
Magner *et al. *(2007) [111]	Liver transplantation	Case report	2	SD-FFP 42 U, 16 U	No control group
Moore *et al. *(2008) [97]	Massive trauma	Prospective noncomparator	93	FFP 6.3 U	No control group
Pull ter Gunne *et al. *(2010) [107]	Surgery (other)	Retrospective noncomparator	300	FFP mean 2.61 U	No control group
Ranucci *et al. *(2008) [98]	CV surgery	Retrospective noncomparator	4546	FFP	No control group
Schols *et al. *(2008) [53]	Surgery (CV, abdominal or spinal bone)	Prospective noncomparator	51	FFP mean 780 ml	No control group
Spinella *et al. *(2008) [99]	Trauma	Retrospective noncomparator	708	FFP mean 3 U	No control group
Stricker *et al. *(2010) [70]	Surgery (other, paediatric)	Retrospective noncomparator	159	FFP mean 20 ml/kg	No control group
Swisher *et al. *(1996) [100]	Surgery (oesophageal resection for cancer)	Retrospective noncomparator	275	FFP	No control group
Tenza *et al. *(2009) [101]	Liver transplantation	Retrospective noncomparator	74	FFP mean 15.7 U	No control group
Watson *et al. *(2009) [103]	Trauma	Retrospective noncomparator	842	FFP median 4.8 U	No control group
Wiederkehr *et al. *(2010) [102]	Liver transplantation	Retrospective noncomparator	155	FFP	No control group
Fibrinogen concentrate					
Bell *et al. *(2010) [112]	Postpartum haemorrhage	Case series	6	Fibrinogen concentrate	No control group
Böhrer (1999) [57]	Liver transplantation	Case report	1	Fibrinogen concentrate 2 g	No control group
Brenni *et al. *(2010) [58]	Trauma	Case report	1	Fibrinogen concentrate 16 g	No control group
Farriols Danés *et al. *(2008) [104]	Various	Retrospective noncomparator	69	Fibrinogen concentrate median 4 g	No control group
Fenger-Eriksen *et al. *(2008) [59]	Severe bleeding	Retrospective noncomparator	43	Fibrinogen concentrate mean 2.0 g	No control group
Glover *et al. *(2010) [63]	Postpartum haemorrhage	Case report	1	Fibrinogen concentrate	No control group
Haas *et al. *(2008) [113]	Surgery (craniofacial, paediatric)	Case series	9	Fibrinogen concentrate median 76 mg/kg	No control group
Heindl *et al. *(2005) [116]	Surgery (other)	Case report	2	Fibrinogen concentrate 7 g, 8 g	No control group
Innerhofer (2006) [60]	Surgery (lumbar)	Case report	1	Fibrinogen concentrate 5 g	No control group
Peitsidou *et al. *(2008) [114]	Emergency caesarean section and Hysterectomy	Case report	1	Fibrinogen concentrate 2 g	No control group
Schöchl *et al. *(2010) [61]	Massive trauma, then laparotomy	Case report	1	Fibrinogen concentrate 13 g	No control group
Schöchl *et al. *(2010) [62]	Trauma surgery	Case report	1	Fibrinogen concentrate 12 g	No control group
Schöchl *et al. *(2010) [73]	Trauma surgery	Retrospective noncomparator	128	Fibrinogen concentrate median 7 g	No control group
Solomon *et al. *(2010) [72]	CV surgery	Retrospective noncomparator	39	Fibrinogen concentrate mean 6.5 g	No control group
Thorarinsdottir *et al. *(2010) [74]	Surgery (various)	Retrospective noncomparator	37	Fibrinogen concentrate median 2 g	No control group
Weinkove *et al. *(2008) [115]	Various	Retrospective noncomparator	30	Fibrinogen concentrate median 4 g	No control group
Other					
Murad *et al. *(2010) [5]	Various	Meta-analysis	12,421	FFP	Various

CV = cardiovascular; FFP = fresh frozen plasma; GI = gastrointestinal; SD-FFP = solvent/detergent-treated fresh frozen plasma.

**Table 4:** Summary of included studies

Study types and interventions	FFP, *n *(%)		Fibrinogen concentrate, *n *(%)	
Study type	*N *= 70		*N *= 21	
RCT		15 (21.4%)		3 (14.3%)
Comparator study		37 (52.9%)		2 (9.5%)
Noncomparator study		18 (25.7%)		16 (76.2%)
Comparison group intervention	*N *= 52		*N *= 5	
Colloid, crystalloid or no intervention		20 (38%)		2 (40%)
Alternative dosage or formulation		32 (62%)		0 (0%)
Fibrinogen concentrate vs FFP		Included in adjacent column		3 (60%)
Meta-analysis	*N *= 1		*N *= 0	

FFP = fresh frozen plasma; RCT = randomised, controlled trial.

The average amount of FFP administered in each study varied greatly, ranging from a nominal prophylactic dose to tens of units (typically in the massive transfusion studies). Several studies did not report the FFP dose administered. The dosage of fibrinogen concentrate typically ranged from 2 to 8 g. In studies of massive transfusion, the intervention group was classified as those patients receiving the highest FFP:RBC ratio (typically 1:1), whereas the control groups comprised those patients who received lower FFP:RBC ratios. Control groups from studies in which the effect of FFP:RBC ratios was not assessed typically received various forms of colloid or crystalloid, apart from those studies directly comparing the administration of fibrinogen concentrate with that of FFP (in addition to other allogeneic components).

### Articles and studies excluded because of overlapping and duplicate data

Studies containing overlapping or duplicate populations were excluded prior to the completion of the data tables. Two articles reported the same data comparing the outcomes of liver transplant patients who were administered either standard FFP or solvent/detergent-treated FFP (SD-FFP) [21,22]. Data from the more recent publication were used [22]. The same study of infants undergoing craniofacial surgery was published in two articles [23,24]. Where the same outcome data were reported in both articles, the earlier publication was used [23]. Two articles also reported data from the same trial of patients undergoing cardiovascular (CV) surgery [25,26]. The article reporting the greater number of outcomes of interest was retained [26].

Three massive transfusion studies reported data from an overlapping population [2,27,28]; however, two of these publications compared cohorts of patients pre- and postimplementation of a massive transfusion protocol. The publication that specifically compared different FFP:RBC ratios was used in this review [28]. Overlapping data were reported in another three massive transfusion studies [4,29,30]. One reported the results from the first year of a prospective study assessing the effect of the FFP:RBC ratio on survival and compared these with matched historical cases [30], the second reported the results from the entire prospective study only [29] and the entire prospective data set was analysed against matched historical cases in the third [4]. Survival data were taken from the most recent publication in which the entire prospective data set was compared against matched historical cases [4]. Additional outcomes of interest were found in the earliest publication, so they were included where appropriate [30]. There were overlapping populations in another three massive transfusion publications [31-33]. For the purposes of this review, they were classified as a single study because subsets of patients were analysed in each publication.

### Blood loss

#### Fresh frozen plasma

Of the comparator studies reporting the effect of FFP on blood loss, one reported lower blood loss as a result of FFP [34]. The investigators in this massive trauma study compared high, medium and low FFP:RBC ratios. Patients receiving a high ratio (1:1.4) of FFP:RBC were less likely than patients in the low ratio group to have uncontrolled early haemorrhage (1:8).

In total, 18 of 20 outcomes indicated no reduction in blood loss (primarily reported as postoperative blood loss) for the FFP group in relation to the comparator group [23,35-50] (Table 5). In one study, 2 of 52 patients who received FFP had 'significant bleeding' > 1,100 ml) versus 0 of 48 in the group that received 5% albumin, though there was no significant difference between the groups in mean blood loss [47]. In another study, the researchers reported a (nonsignificant) trend towards increased chest tube drainage (CTD) in the FFP group compared with the control group that received prothrombin complex concentrates [37]. In addition, in one study of paediatric cardiopulmonary bypass surgery patients, patients who weighed > 8 kg showed significantly greater 24-hour CTD volume following administration of FFP than those who did not receive FFP [44].

**Table 5:** Evidence for the effect of each intervention on blood loss

	FFP outcomes: blood loss			Fibrinogen concentrate outcomes: blood loss		
	
Study type	Reduction	No difference	Increase	Reduction	No difference	Increase
RCT	-	2 (intraoperative) [43,45] 9 (postoperative) [35-38,41,45,48-50]	-	1 (postoperative) [54]	1 (postoperative) [56]	-
Non RCT or prospective comparator	-	2 (postoperative) [23,44]	1 (postoperative) [44]	1 (postoperative) [55]	-	-
Retrospective comparator	1 (perioperative) [34]	2 (intraoperative) [39,40] 2 (postoperative) [46,47] 1 (perioperative) [42]	-	1 (postoperative) [26]	-	-
Controlled and/or comparator study outcomes, *n *(percentage of all outcomes within intervention group)	1 (5%)	18 (90%)	1 (5%)	3 (75%)	1 (25%)	0
Prospective noncomparator	-	1 (postoperative) [53]	-	-	-	-
Retrospective noncomparator	-	1 (postoperative) [52]	-	1 (perioperative) [59]	-	-
Case report	-	1 (perioperative) [51]	-	6 (perioperative) [57,58,60-63]	-	-
Study outcomes, *n *(percentage of outcomes within intervention group)	1 (4%)	21 (92%)	1 (4%)	10 (91%)	1 (9%)	0

FFP = fresh frozen plasma; RCT = randomised, controlled trial; - = no data.

All three noncomparator trials that were identified reported no reduction in blood loss following administration of FFP [51-53]. Two studies, one block-RCT [38] (evaluating the effectiveness of FFP versus 'pathogen-reduced' plasma) and one prospective observational study [53], showed mixed results following administration of FFP. In the RCT, the efficacy of FFP was reported as 'good' (defined as the infusion causing arrest or > 50% reduction in CTD within a six-hour period) in 42% of patients; however, it was judged to have had no influence on the amount of blood lost in 23% of patients [38]. There was no significant difference between the FFP and 'pathogen-reduced' plasma groups. In the observational study, 37% of patients continued to bleed after FFP administration [53]. Interestingly, the plasma fibrinogen level, as well as its increase, after transfusion was markedly lower in patients with ongoing bleeding than in those who stopped bleeding, suggesting that the amount of fibrinogen delivered by the administration of FFP (mean ± SD = 780 ± 280 ml) was not sufficient to achieve haemostasis in a subset of patients.

#### Fibrinogen concentrate

Four comparator studies reported the effect of fibrinogen concentrate on blood loss (Table 5). Three of these studies involved adults, of which one was an RCT involving the prophylactic administration of 2 g of fibrinogen concentrate prior to surgery [54]. The other two were comparator trials evaluating the post cardiopulmonary bypass administration of 7.8 ± 2.7 g [55] and 5.7 ± 0.9 g [26] fibrinogen concentrate compared with approximately 8 U of FFP (+PC) in patients with ongoing bleeding. All three studies showed a positive effect of fibrinogen concentrate administration, which reduced postoperative blood loss by 32 to 59% compared with the control group, which received 'standard' allogeneic transfusion therapy in two cases [26,54,55]. Another RCT investigated the effect of fibrinogen concentrate administration and transfusion therapy guided by thromboelastography compared with 'standard allogeneic transfusion therapy' in children undergoing complex CV surgery [56]. Postoperative blood loss at 1, 6 and 24 hours by CTD was similar in both groups.

Researchers in another seven noncomparator articles (one retrospective observational study and six case studies) observed a beneficial effect of fibrinogen concentrate on blood loss, with haemostasis frequently achieved after allogeneic transfusion therapies had failed [57-63]. The retrospective observational study of 43 patients (comprising 39 adults, 8 of whom were excluded from analysis because of early death, and 4 newborns) reported a significant decrease in median blood loss (from 4,000 to 50 ml) in the adult patients following administration of fibrinogen concentrate during serious haemorrhage [59]. A case report of a trauma patient suggested that 15 U of FFP would have been required to achieve haemostasis in place of the 5-g fibrinogen concentrate administered, which would have exposed the patient to the risk of volume overload as well as the risks associated with administration of any allogeneic blood product [60].

### Allogeneic transfusion requirements

#### Fresh frozen plasma

A total of 19 comparator studies reported 22 outcomes of the effect of FFP on allogeneic transfusion requirements [22,23,30,35-37,39,41,43,44,47,49,50,64-69]. These requirements were reported as intraoperative, postoperative or total (studies in which the requirements were reported for the whole perioperative period). Of these studies, only five showed some benefit for FFP over the comparator group, with the majority of the studies finding no effect and two studies reporting an increase in allogeneic transfusion requirements in the groups receiving FFP (Table 6).

**Table 6:** Evidence for the effect of each intervention on allogeneic transfusions

	FFP outcomes: allogeneic transfusions			Fibrinogen concentrate outcomes: allogeneic transfusions		
	
Type of study	Reduction	No difference	Increase	Reduction	No difference	Increase
RCT	-	3 (postoperative) [35-37] 6 (perioperative) [22,41,43,49,64,65]	1 (intraoperative) [50]; 1 (postoperative) [50]	2 (postoperative) [56,71]	2 (intraoperative) [56,71] 1 (postoperative) [54]	-
Non RCT or prospective comparator	-	1 (intraoperative) [23] 1 (postoperative) [23]	1 (postoperative) [44]	1 (postoperative) [55]	-	-
Retrospective comparator	1 (postoperative) [39] 1 > 24 hours postoperative) [30] 3 (total) [66-68]	1 (postoperative) [47] 2 ≤ 24 hours postoperative) [30,69]	-	1 (intraoperative) [26] 1 (postoperative) [26]	-	-
Controlled and/or comparator study outcomes, *n *(percentage of all outcomes within intervention group)	5 (23%)	14 (63%)	3 (14%)	5 (63%)	3 (37%)	0
Prospective noncomparator	-	1 (postoperative) [53]	-	-	-	-
Retrospective noncomparator	1 (postoperative) [70]	-	-	2 (intraoperative) [72,73] 3 (postoperative) [59,72,74] 1 (perioperative) [73]	-	-
Case report	-	-	-	5 (perioperative) [57,58,60-62]	-	-
Study outcomes (percentage of outcomes, *n *(within intervention group)	6 (25%)	15 (63%)	3 (12%)	16 (84%)	3 (16%)	0

FFP = fresh frozen plasma; RCT = randomised, controlled trial; - = no data. Allogeneic products are defined as any combination of packed red blood cells, FFP, platelets and/or cryoprecipitate.

Seven studies investigated the effect of FFP compared with a non blood product (for example, colloid and/or crystalloid). Six of these showed no difference in the intraoperative [23], postoperative [23,35,36,47] or total [41,49] requirements (requirements were measured as either RBC or any allogeneic product and excluded the study dose of FFP). A block-RCT of coronary artery bypass graft surgery patients compared those who received 4 U of FFP intraoperatively with controls who received hydroxyethyl starch (HES) [50]. The study showed that transfusion requirements for RBC were significantly higher (both intra- and postoperatively) among those patients receiving FFP compared with patients administered HES. A further (paediatric) study also found that a greater proportion of patients in the FFP group required postoperative transfusions compared with those not receiving FFP [44]. Two studies that compared FFP with 'pathogen-reduced' plasma showed no differences between groups in the total requirements for allogeneic transfusion [22,65].

There were six articles in which the effect of the FFP:RBC ratio on transfusion requirements was assessed. Three found reduced RBC requirements at higher FFP:RBC ratios (typically 1:1 to 1:1.5) [66-68]. At FFP:RBC ratios ≥ 1:2 versus < 1:2, there was no difference in RBC requirements over the initial 6 or 24 hours; however, the longer-term > 24 hours) transfusion requirements for both RBC and FFP were lower in the higher ratio group [30]. One further study (of low sample size) also demonstrated that postoperative RBC, FFP and PC requirements were reduced by increasing the amount of FFP administered intraoperatively to achieve a FFP:RBC ratio closer to 1:1 [39].

There were another two noncomparator studies (one prospective and one retrospective) reporting the effect of FFP on allogeneic transfusion requirements [53,70]. The volume of intraoperatively transfused FFP was inversely associated with the postoperative administration of allogeneic products in one (paediatric) study [70]. There was a mixed response to FFP in the other study; 2 U were sufficient to achieve haemostasis in 63% of patients, while the remaining 37% received additional transfusions [53].

#### Fibrinogen concentrate

The effect of fibrinogen concentrate on allogeneic transfusions was assessed in five comparator trials, four of which showed a reduction in requirements as a result of the intervention [26,54-56,71] (Table 6). Notably, the exposure to all allogeneic products (RBCs, FFP and PC) transfused postoperatively was reduced in two of these studies [26,55]. In the control groups (treated with approximately 8 U of FFP), 100% of patients received postoperative transfusions compared with only 20% [55] and 44% [26] of patients who received fibrinogen concentrate. A third study reported lower postoperative RBC requirements in the fibrinogen concentrate group compared with the control group (that received saline), with no difference found for intraoperative RBC requirements [71]. The fourth study, a RCT of children undergoing CV surgery, found a significant reduction in postoperative and total FFP requirements in the fibrinogen concentrate group compared with the control group [56]. The intervention group received less than half the amount of FFP than the control group (10.6 ± 6.5 ml/kg versus 22.5 ± 13.1 ml/kg). No differences were seen in the amount of intra-, post- or perioperative RBC administered. A fifth study reported no difference in the amount of postoperative allogeneic products administered to the group that had received a prophylactic dose of 2 g of fibrinogen concentrate prior to surgery compared with the control group (no fibrinogen concentrate) [54].

Four retrospective noncomparator studies provided evidence for a reduction in allogeneic transfusion requirements following administration of fibrinogen concentrate, with a further five case studies also suggesting a reduction as a result of the intervention [57-62,72-74]. One of the noncomparator studies that presented data derived from CV surgery patients reported that 35 of 39 patients did not receive any further intraoperative FFP or PC after administration of fibrinogen concentrate (the remaining four patients received PC) [72]. In addition, in the postoperative period, only 11 of 39 patients required additional transfusions, whereas in another sizable study of trauma patients, only 6 of the 123 patients who received fibrinogen concentrate were administered intraoperative FFP, and only 12 of 128 received FFP in the first 24 hours after admission to the emergency room [73]. The other two noncomparator studies demonstrated a reduction in allogeneic transfusions, with one reporting a drop in transfusion of RBC from 6 U to 3 U after administration of fibrinogen concentrate [74] and the other reporting significantly fewer allogeneic transfusions in the 24 hours after administration of the fibrinogen concentrate than in the 24 hours prior to administration [59].

### Survival

#### Fresh frozen plasma

We found 32 comparator studies involving FFP reporting results from 40 time points over periods ranging from 6 hours to 10 years [3,4,28,31-34,38-40,42,66-69,75-93] (Table 7). Eight studies compared the effect of FFP with no FFP, of which, three showed no effect of the intervention on survival (trauma with nonmassive transfusion [79], hepatectomy for hepatocellular carcinoma [40] and CV surgery [84]), and five reported reduced survival for patients receiving FFP (trauma [77], traumatic brain injury [78], liver transplantation [82], liver resection for colorectal metastases [88] and hepatectomy for hepatocellular carcinoma [92]). In the trauma study, the investigators found a dose-dependent correlation between FFP transfusion and mortality, with each unit of FFP given increasing the risk of death by 3.5% [77].

**Table 7:** Evidence for the effect of each intervention on survival

	FFP outcomes: survival			Fibrinogen concentrate outcomes: survival		
	
Type of study	Improvement	No difference	Reduction	Improvement	No difference	Reduction
RCT	-	-	1 (1 to 32 days) [38] 1 (in-hospital) [78]	-	-	-
Non RCT or prospective comparator	-	1 (in-hospital) [87]	1 (in-hospital) [77]	-	^-^	-
Retrospective comparator	2 (6 hours) [68,81] 3 (24 hours) [4,67,81] 7 (30 days) [3,4,28,39,42,69,81] 1 (90 days) [69] 7 (in-hospital) [31-34,67,68,86,90,91]	4 (30 days) [66,84,85,93] 1 (36 months) [40] 6 (in-hospital) [31,67,76,79,83,89]	1 (30 days) [92] 1 (1 year) [82] 1 (10 years) [88] 2 (in-hospital) [75,80]	-	1 (30 days) [26]	-
Controlled and/or comparator study outcomes, *n *(percentage of all outcomes within intervention group)	20 (50%)	12 (30%)	8 (20%)	0	1 (100%)	0
Prospective noncomparator	1 (24 hours) [97]	-	1 (in-hospital) [95]	-	-	-
Retrospective noncomparator	2* (in-hospital) [99,103]	1 (6 months) [101] 1 (1 year) [102] 1 (68 months) [100] 2* (in-hospital) [96,103]	1 (at 6 months) [101] 2 (in-hospital) [94,98]	1 (7 days) [104] 1 (in-hospital) [73]	-	-
Case report	-	-	-	-	-	-
Study outcomes, *n *(percentage of outcomes within intervention group)	23 (44%)	17 (33%)	12 (23%)	2 (67%)	1 (33%)	0

FFP = fresh frozen plasma; RCT = randomised, controlled trial; - = no data. *Primary analysis excluding early deaths < 48 hours) revealed no association with FFP administration and survival. Secondary analysis including all patients revealed an association with decreased mortality [103].

The effect of FFP on patient mortality has largely been reported in the form of articles exploring the impact of different FFP:RBC ratios, of which we found 19 such studies. Sixteen studies reported the impact of FFP:RBC ratios in trauma patients, typically by comparing a prospective cohort that received a 'transfusion protocol' with a historical cohort that received treatment prior to implementation of the protocol [3,4,28,31-34,66-68,80,81,85-87,89-91]. In general, outcomes were favourable for patients who received more FFP than for those who received less FFP, though the exact ratio needed to achieve an improvement in the survival rate was not consistent. In fact, one study suggested that the survival rate followed a U-shaped curve and that the lowest predicted mortality was associated with a FFP:RBC ratio of 1:2 to 1:3, with a FFP:RBC ratio of 1:1 resulting in reduced survival [80].

Murad and colleagues [5] conducted a meta-analysis of studies reporting of the effect of FFP on mortality compared with a control group and found that, for patients undergoing massive transfusion > 10 U of RBC), FFP:RBC ratios in the range of 1:2.5 to 1:1 were associated with a significant reduction in mortality risk (OR = 0.38). However, this improvement in survival was reversed in surgical patients who did not receive massive transfusions, where FFP was associated with a trend towards increased mortality (OR = 1.22).

Ten noncomparator studies also reported on the effect of FFP on survival [94-103]. Both studies of CV surgery patients showed a significant association between FFP and reduced in-hospital survival (OR = 2.51 and OR = 12.6) [94,98]. In one trauma study, FFP was shown to have no effect on mortality when early deaths < 48 hours) were excluded. Furthermore, FFP administration was associated with a 2.9% decreased risk of mortality for every unit transfused when the early deaths were included [103]. Higher levels of FFP were also reported to improve survival in two trauma studies [97,99]. Another study found no significant association between FFP administered in the ICU and in-hospital survival rates (OR = 1.03) [96].

#### Fibrinogen concentrate

Only three fibrinogen concentrate studies, one retrospective comparator study and two retrospective noncomparator studies [26,73,104] (Table 7), were suitable for inclusion in this section of the review. There were no deaths reported in either arm of the fibrinogen concentrate studies included elsewhere in this review. In the retrospective comparator study, there was no significant difference in 30-day mortality between groups. In the 'standard therapy' group, 2 (17%) of 12 patients died within 30 days of surgery compared with 0 (0%) of 6 patients in the fibrinogen concentrate group, although this difference was nonsignificant (*P *> 0.05).

Analysis by logistic regression of data from patients with acquired acute afibrinogenaemia (due to surgery and/or trauma) in one retrospective noncomparator study indicated a statistically significant association between the higher plasma fibrinogen levels measured after fibrinogen concentrate administration and 7 day patient survival [104]. The other study, which reported the effect of fibrinogen concentrate administration to trauma patients, observed significantly reduced mortality compared with the mortality predicted by Trauma Injury Severity Score (TRISS) and by revised injury severity classification (RISC) [73].

### Length of stay

#### Fresh frozen plasma

The impact of FFP on LOS for patients in the ICU was reported in 14 comparator studies [3,24,32,33,39,50,66,67,69,77,79,81,83,87,105] (Table 8). Of these, over half reported an increased ICU LOS for patients in the (high) FFP group compared with controls [39,66,67,69,77,81,83,105], with two studies reporting ICU stays were over 50% longer for patients administered FFP compared with the nontransfused controls [77,105]. Conversely, in one study, a 1:1 FFP:RBC ratio was associated with an approximately 50% reduction in ICU LOS compared with a 1:4 ratio [32,33]. Three studies found no difference in ICU LOS between the group that received FFP and a control group that did not [24,50,79].

**Table 8:** Evidence for the effect of each intervention on ICU and/or hospital length of stay

	FFP outcomes: ICU and/or hospital length of stay			Fibrinogen concentrate outcomes: ICU and/or hospital length of stay		
	
Type of study	Reduced	No difference	Increased	Reduced	No difference	Increased
RCT	-	1 (ICU) [50]	-	1 (ICU) [56] 1 (hospital) [56]	-	-
Non RCT or prospective comparator	1 (hospital) [24]	2 (ICU) [24,87] 1 (hospital) [87]	1 (ICU) [77] 1 (hospital) [77]	1 (ICU) [55]	1 (hospital) [55]	-
Retrospective comparator	1 (ICU) [32,33]	2 (ICU) [3,79] 5 (hospital) [3,39,40,66,79]	7 (ICU) [39,66,67,69,81,83,105]* 3 (hospital) [67,69,81]	1 (ICU) [26]	1 (hospital) [26]	-
Controlled and/or comparator study outcomes, *n *(percentage of all outcomes within intervention group)	2 (8%)	11 (44%)	12 (48%)	4 (67%)	2 (33%)	0
Prospective noncomparator	-	-	-	-	-	-
Retrospective noncomparator	-	1 (ICU) [107] 1 (hospital) [107]	2 (ICU) [98,106]	-	-	-
Case report	-	-	-	-	-	-
Study outcomes, *n *(percentage of outcomes within intervention group)	2 (7%)	13 (45%)	14 (48%)	4 (67%)	2 (33%)	0

FFP = fresh frozen plasma; RCT = randomised, controlled trial; - = no data. *For Oberkofler 2010 [83], this represented an increased risk of ICU stay > 10 days.

Eleven comparator studies reported the effect of FFP on patients' hospital LOS [3,24,39,40,66,67,69,77,79,81,87], with only one reporting a benefit for FFP [24] (Table 8). Of the three retrospective noncomparator studies involving FFP, administration was significantly associated with an increased or prolonged ICU stay in two [98,106], but it was not correlated with LOS in the third [107]. Hospital LOS was reported in one noncomparator study in which surgical patients were analysed. That study found that, as with ICU LOS, FFP use was not correlated with hospital LOS [107].

#### Fibrinogen concentrate

All three fibrinogen concentrate studies reporting LOS showed significant reductions in the time spent in the ICU of 11 hours [55], 78 hours [26] and 36 hours [56] for patients in the intervention group compared with those in the control (FFP) group. Hospital LOS was also reported in these three studies. One observed a significantly reduced hospital LOS for patients in the fibrinogen concentrate group compared with the control group (21 days versus 32 days, respectively) [56]. The other two studies found no difference in hospital LOS between patients in the intervention and control groups [26,55].

### Plasma fibrinogen levels

#### Fresh frozen plasma

Eleven FFP comparator studies reported plasma fibrinogen levels pre- and postadministration or in relation to a control [22,23,30,38,41,43,44,49,64,108,109] (Table 9). It is difficult to compare most of the results directly because they differ widely in the way in which they were reported. For instance, some studies compared plasma fibrinogen levels pre- and postadministration, whereas others compared the difference in levels between groups at certain time points. To further complicate matters, many comparisons were made between groups of patients receiving different doses or formulations of FFP and did not compare the effect of FFP against a non plasma product. A positive effect was seen for the FFP group in five studies [23,30,38,41,43] whereas the control group had higher levels in two [44,108].

**Table 9:** Evidence for the effect of each intervention on plasma fibrinogen levels

	FFP outcomes: plasma fibrinogen levels			Fibrinogen concentrate outcomes: plasma fibrinogen levels		
	
Type of study	Increased/higher	No difference	Decreased/lower	Increased/higher	No difference	Decreased/lower
RCT	2 (vs preadministration) [38,43] 1 (vs comparator) [41]	3 (vs preadministration/comparator) [22,49,64]	1 (vs preadministration) [108]	2 (vs comparator) [54,71]	-	-
Non RCT or prospective comparator	1 (vs comparator) [23]	1 (vs preadministration/comparator) [109]	1 (vs comparator) [44]	1 (vs comparator) [55]	-	-
Retrospective comparator	1 (vs comparator) [30]	-	-	1 (vs comparator) [26]	-	-
Controlled and/or comparator study outcomes, *n *(percentage of outcomes within intervention group)	5 (46%)	4 (36%)	2 (18%)	4 (100%)	0	0
Prospective noncomparator	1 (vs preadministration) [53]	-	-	-	-	-
Retrospective noncomparator	1 (vs preadministration) [70]	-	1 (vs preadministration) [110]	6 (vs preadministration) [59,72-74,104,115]	-	-
Case report	-	-	2 (vs preadministration) [51,111]	7 (vs preadministration) [60-62,112-114,116]	-	-
Study outcomes, *n *(percentage of outcomes within intervention group)	7 (44%)	4 (25%)	5 (31%)	17 (100%)	0	0

FFP = fresh frozen plasma; RCT = randomised, controlled trial; - = no data.

Four studies assessed the effect of FFP versus a non blood product. Two found significantly higher plasma fibrinogen levels postadministration in the FFP group than in the control group [23,41]. Intraoperative plasma fibrinogen levels were maintained at the preoperative level of 2.0 g/L in the FFP group compared with a decline to an intraoperative value of 1.3 g/L in the albumin group in the study involving infants undergoing craniofacial surgery [23]. In the study of adults undergoing CV surgery, plasma fibrinogen levels fell from a baseline value of 2.7 g/L to a postadministration value of 1.8 g/L in the FFP group compared with a fall from 2.9 g/L to 1.3 g/L in the HES group, at which point plasma fibrinogen levels were significantly higher in the FFP group than in the control group [41]. A third study reported significantly reduced levels from baseline values throughout the operative period, with no significant differences between the two study groups at any point despite the administration of 600 ml of FFP to one group [49]. The fourth study reported significantly lower plasma fibrinogen levels compared with baseline in the FFP group, but not in the control group [108].

Chowdhury and colleagues [109] reported the effect on plasma fibrinogen levels of two different doses of FFP (12.2 ml/kg and 30 ml/kg) in a prospective, observational study of a consecutive cohort of patients with haemorrhage in the ICU. The first 10 patients received the lower dose, and the next 12 patients received the higher dose. There was no significant difference between the two groups postadministration, and plasma fibrinogen levels were not significantly higher postadministration than preadministration in either group.

Five noncomparator studies reported the effect of FFP on plasma fibrinogen levels. Two indicated a benefit from FFP [53,70], whilst three found that FFP was associated with lower plasma fibrinogen levels [51,110,111]. Of note, one study reporting the effect of FFP on plasma fibrinogen levels in a cohort of paediatric surgery patients found the volume of FFP was inversely associated with a postoperative plasma fibrinogen level < 1.0 g/L [70].

#### Fibrinogen concentrate

Plasma fibrinogen levels following administration of fibrinogen concentrate were reported in four comparator studies [26,54,55,71] (Table 9). In all studies, plasma fibrinogen levels were significantly higher postadministration in the fibrinogen concentrate group than in the control group (which received FFP in two studies). Increases in plasma fibrinogen levels postadministration ranged from 0.6 g/L following a preoperative 2-g prophylactic dose [54] to 2.0 g/L following a mean intraoperative dose of 7.8 g [26]. Importantly, plasma fibrinogen levels were not significantly higher in the fibrinogen concentrate group than in the control group at the next assessment point (6 to 24 hours later).

A further 13 noncomparator studies presented data on the effect of fibrinogen concentrate on plasma fibrinogen levels, all of which also showed an increase in plasma fibrinogen levels after the intervention [59-62,72-74,104,112-116]. Six retrospective observational studies showed mean increases in plasma fibrinogen levels, ranging from 0.6 g/L after a median dose of 2 g [74] to 1.7 g/L after a mean dose of 6.5 g [72].

## Discussion

### Quality of evidence

There is a relative paucity of high-quality evidence reporting the outcome of administration of FFP in a perioperative or massive trauma setting, despite the long period of its usage. Also, few high-quality trials were identified that reported the outcome of administration of fibrinogen concentrate perioperatively, and none assessed the intervention during massive trauma. Controlled trials with robust blinding and randomisation procedures are the gold standard when assessing the efficacy and safety of interventions. However, the low number of RCTs conducted for each intervention may reflect the difficulties in designing and implementing such trials in bleeding patients who are in potentially life-threatening situations.

Despite there being fewer fibrinogen concentrate comparator studies than FFP comparator studies (5 versus 52, respectively), the nature of the comparison must also be considered. Of the FFP comparator studies analysed, only 38% (20 of 52) compared FFP with no FFP or with a non blood product (for example, colloid or crystalloid). Many FFP studies assessed outcomes against a control group that received a different dosage or formulation of FFP reflecting the widely held assumption that 'standard' FFP is efficacious in these situations. In contrast, 40% (2 of 5) of fibrinogen concentrate studies compared the intervention with either no fibrinogen concentrate or a colloid or crystalloid control, and the other 60% (3 of 5) compared the use of fibrinogen concentrate with FFP. Therefore, the low number of comparator trials for fibrinogen concentrate still provided useful evidence and allowed valuable comparisons to be made between the intervention and a non blood control, as well as a direct comparison with FFP. Whilst observations can be made about the relative efficacies of FFP and fibrinogen concentrate in the perioperative setting, it is notable that, despite a number of noncomparator studies of fibrinogen concentrate in the trauma setting (with one recent study involving 128 patients), there are no comparator studies involving the use of fibrinogen concentrate in patients with haemorrhage due to massive trauma. This lack of evidence highlights a need for more research in this area so that the efficacy of fibrinogen concentrate can be assessed in this clinical setting.

#### Potential for bias

The majority of studies included in this review were observational in nature and as such could be subject to bias, particularly in studies in which researchers were looking for associations between outcomes and different dosages of an intervention. Most of the studies did not report whether they had performed an assessment of, or had controlled for, bias when calculating the effect of the intervention in question. Bias may have occurred by a number of mechanisms. First, a selection bias may have been in effect because the most resources were directed towards the patients deemed most likely to survive. Second, in other studies, the less-well patients may have received more of an intervention because they were more ill. Third, the studies may have had a survival bias where patients in the worst condition died too quickly to receive a high dose of the intervention, so, by default, the patients with a poor prognosis were preferentially included in the low-dose groups. In these instances, data suggesting that the intervention was responsible for a difference in morbidity and/or mortality between groups may not be reliable. It is possible that the intervention group had a prognosis different from that of the comparison group *regardless *of the amount of the intervention administered, *post hoc ergo propter hoc*.

The use of fibrinogen concentrate as a haemostatic intervention in the management of perioperative bleeding is still in its early years. Therefore, in the current literature, there may be a publication bias towards studies demonstrating the successful use of a product rather than those reporting failure.

### Efficacy outcomes

**Figure 2:**
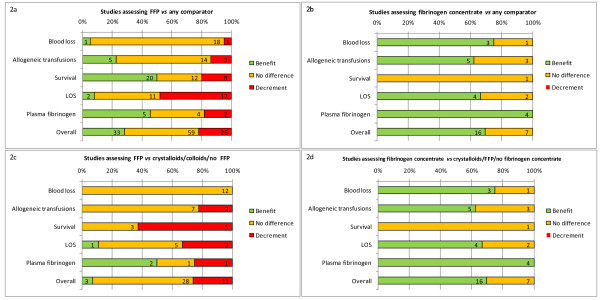
**Summary of efficacy outcomes from comparator trials**. Numbers represent number of outcomes. FFP = fresh frozen plasma; LOS = length of stay.

Our systematic literature search identified 70 FFP and 21 fibrinogen concentrate publications reporting the effects of the intervention on a number of outcomes of interest. However, although 74% of identified FFP studies had a comparator group, many involved different doses or formulations of FFP rather than a non FFP arm. Furthermore, the majority of fibrinogen concentrate studies (76%) were of low quality and did not report the effect of the intervention in relation to a comparator group. Figure 2 summarises the results of all studies in our review with a comparator group for each intervention by reported outcome and also as a combined total of all outcomes. Figure 2a reinforces the doubt about the efficacy of FFP, assessed across a range of outcomes, with no single outcome reflecting a benefit for FFP in > 50% of extracted measures. When all outcomes were added together, a benefit of FFP administration was found in only 28% of measures, only slightly more than those reporting a negative effect of FFP (22%). If only those studies comparing FFP with a non FFP group are considered, the picture is even less convincing, because only 7% of reported outcomes supported a beneficial effect of FFP (Figure 2c). In contrast, the evidence for the efficacy of fibrinogen concentrate was far more consistent, with no negative outcomes reported for any measure (Figure 2b). Fibrinogen concentrate was shown to reduce blood loss, reduce allogeneic transfusion requirements, reduce ICU and hospital LOS and increase plasma fibrinogen levels in over two-thirds of reported outcomes. In the five comparator trials, 70% of outcomes showed a benefit of fibrinogen concentrate over the control. Importantly, the control was FFP in three of the studies, thus providing some evidence that fibrinogen concentrate is more efficacious than FFP across a range of clinical outcomes in the perioperative setting.

The strongest support for a benefit for FFP derives from studies reporting survival, where 50% suggested that FFP (typically at higher FFP:RBC ratios) reduces mortality; however, FFP was associated with increased mortality in 20% of studies. In general, studies reporting an association of higher doses of FFP with improved survival assessed the effect of FFP:RBC ratios during massive transfusion. This finding is in agreement with the meta-analysis performed by Murad and colleagues [5]. Many studies targeting higher FFP:RBC ratios did so by increasing the amount of FFP administered in the early phase of massive haemorrhage. This temporal aspect of FFP administration was highlighted in a recent publication which found that patients who received an early high FFP:RBC ratio were in less severe shock and less likely to die early from uncontrollable haemorrhage than were those patients in the low FFP:RBC ratio group, who never achieved a high ratio [117]. The survival advantage associated with the higher FFP:RBC ratios currently being lauded in the literature may be due partly to selection, whereby patients in such studies die *with *a low FFP:RBC ratio, *not because of *a low ratio. Fibrinogen deficiency manifests early in bleeding patients. It is possible that an improvement in survival rates at higher FFP:RBC ratios was due in part to earlier supplementation of plasma fibrinogen in the resuscitation effort and not to a benefit of FFP *per se*. The fibrinogen concentrate studies identified were typically small, with a mean of only 10 patients per arm. Consequently, there were almost no deaths reported in either group, making a robust assessment of any survival benefit following the administration of fibrinogen concentrate (early or late) virtually impossible.

In this review, we examined relevant outcomes of interest by analysing the literature regarding one of two interventions: FFP and fibrinogen concentrate. However, haemostatic support during surgery or massive trauma is rarely achieved by the administration of one product alone; therefore, the majority of the studies included in this review involved the administration of other products, particularly RBC, but also PC, cryoprecipitate, prothrombin complex concentrate, tranexamic acid, aprotinin and others. The influence of coadministered products on the outcomes of interest was not studied in this review, though the potential for an impact should be considered when drawing any conclusions regarding the impact of each intervention on these outcomes, particularly in studies where cryoprecipitate was administered, as this would provide a more concentrated dose of fibrinogen than FFP alone.

### Risk versus benefit

The benefits of any intervention should outweigh the risks. In this review, we found inconsistent and contradictory evidence concerning the efficacy of FFP. Furthermore, the findings of this review are in line with other published work, where FFP has particularly been associated with an increased risk of mortality when used during nonmassive transfusion [5]. In terms of risks, Murad and colleagues examined the incidence of ALI and MOF in their meta-analysis [5], in which they reported that FFP significantly increased the risk of pulmonary complications (OR = 2.92). Contrary to researchers in other studies [103,118], however, they reported a significant reduction in the risk of MOF with FFP administration (OR = 0.40). Although the risk of viral and bacterial transmission by FFP exists, it is very low. The introduction of nucleic acid screening for known infectious diseases has led to the transmission of infectious diseases being rare [119]. Other adverse events (AEs) are associated with the use of FFP, such as allergic and haemolytic transfusion reactions, but these are infrequent ≤ 1 event per 100,000 blood components issued [6]). Furthermore, several strategies have been employed successfully to reduce many of the risks associated with 'standard' FFP, such as the introduction of different formulations of plasma (for example, SD-FFP and photochemical treatment FFP, lyophilised and others), the use of leucocyte-depleted plasma and restricting the use of FFP from female donors [9]. Half of all outcomes analysed in this review indicated that FFP had no effect, positive or negative, and it could be argued that administration of FFP is worthwhile on the basis of the possibility that it might be efficacious and at worst will effect no change in clinical parameters. Nonetheless, when the potential risks associated with FFP, however rare, are considered in the context of the efficacy findings in this study, in which fewer than one-third of the reported outcomes favoured FFP over the comparator, the continued use of the product should be questioned in an approach that weighs risk versus benefit.

There was consistent evidence that fibrinogen concentrate improved the outcomes studied in this review. More than two-thirds (16 of 23) of all outcomes showed a benefit of fibrinogen concentrate over any comparator, notably 72% (13 of 18) of the outcomes were favourable for fibrinogen concentrate over FFP. Furthermore, no study reported a negative effect versus a comparator on any outcome measure included in this review. In terms of the risks involved with fibrinogen concentrate, there is a low risk of AEs such as allergic reactions, and, in rare cases, administration of fibrinogen concentrate has been associated with thromboembolic events [120,121]. A number of preclinical studies have provided evidence supporting the safety and tolerability of fibrinogen concentrate, and preclinical models, including one of venous stasis, have shown no evidence of thrombosis formation in treated animals, demonstrating the low thrombogenic potential of the product [120,122-125]. In addition, a pharmacosurveillance report and systematic review of thrombotic events in clinical studies (of patients with both congenital and acquired afibrinogenaemia) showed no significant safety concerns associated with fibrinogen concentrate (Haemocomplettan P; CSL Behring, Marburg, Germany) use in perioperative bleeding situations with regard to thrombogenicity [120]. Over a 22-year pharmacosurveillance period, nine thrombotic events possibly related to the administration of fibrinogen concentrate were reported (seven of which were in patients with congenital fibrinogen deficiency) at an incidence of 3.48 per 100,000 treatment episodes. However, further safety studies employing rigorous methods intended to detect conditions such as deep vein thrombosis are still required to confirm the findings from the preclinical and pharmacosurveillance studies.

Among the trials included in this review [26,54-63,71-74,104,112-116], there was a low incidence of the predefined safety outcomes (thrombotic events, ALI, TACO, infections [bacterial contamination and viral transmission] and MOF). Furthermore, there was a low frequency of AEs of any nature in the 17 fibrinogen concentrate studies in which AEs were reported (Table 10). Of the trials with a comparator group, an AE (postoperative atrial fibrillation) was reported for 1 of 36 (3%) patients receiving fibrinogen concentrate compared with 7 of 37 (19%) of patients in the comparator groups [26,54,55,71]. There were another seven AEs reported in the noncomparator trials and case reports of 240 patients receiving fibrinogen concentrate (3%) [57-61,63,72,74,104,112,113,115,116]. Four of the AEs (all arterial ischaemic events) occurred between 4 and 12 days (median = 7.5 days) after fibrinogen concentrate administration in postoperative surgical patients who had massive perioperative haemorrhage and required more than 12 U of RBC [115]. Of the remaining three AEs, one case of 'jitter and snoring respiration' was reported by nursing staff, though the patient was judged to be alert with normal respiration upon the arrival of the attending physician; one patient complained of attacks of shivering 24 hours after fibrinogen concentrate administration [59]; and one patient who received massive transfusion of a variety of haemostatic products had subsequent acute renal failure [116].

**Table 10:** Adverse events reported in fibrinogen concentrate trials

		Patients, *N*		Adverse events*^, *n *(%)		
			
Study type	Indication	Fibrinogen concentrate	Comparator	Fibrinogen concentrate	Comparator	Details
RCTs						
Cui *et al. *(2010) [56]	CV surgery (children)	17	14	-	-	Adverse events not reported
Fenger-Eriksen *et al.* (2009) [71]	Surgery (cystectomy)	10	10	0	0	
Karlsson *et al. *(2009) [54]	CV surgery	10	10	0	1 (10%)	1 perioperative myocardial infarction (comparator group)
Trials with a comparator group						
Rahe-Meyer *et al.* (2009) [55]	CV surgery	10	5	1 (10%)	1 (20%)	2 postoperative atrial fibrillation (1 fibrinogen concentrate group, 1 comparator group)
Rahe-Meyer *et al.* (2009) [26]	CV surgery	6	12	0	5 (42%)	1 postoperative atrial fibrillation (comparator group) 2 renal failure (2 comparator group) 2 major neurological events (2 comparator group)
Totals (comparator trials reporting adverse events)		36	37	1 (3%)	7 (19%)	
Noncomparator trials and case reports						
Bell *et al. *(2010) [112]	Postpartum haemorrhage	6	-	0	-	
Böhrer *et al. *(1999) [57]	Liver transplantation	1	-	0	-	
Brenni *et al. *(2010) [58]	Trauma	1	-	0	-	
Farriols Danés *et al.* (2008) [104]	Various	69	-	0	-	
Fenger-Eriksen *et al.* (2008) [59]	Severe bleeding	43	-	2 (5%)	-	1 jitter and snoring respiration 1 shivering (causal relationship to fibrinogen concentrate administration could not be excluded in either case)
Glover *et al. *(2010) [63]	Postpartum haemorrhage	1	-	0	-	
Haas *et al. *(2008) [113]	Surgery (craniofacial, paediatric)	9	-	0	-	
Heindl *et al. *(2005) [116]	Surgery (other)	2	-	1	-	1 acute renal failure (case 1)
Innerhofer (2006) [60]	Surgery (lumbar)	1	-	0	-	
Peitsidou *et al. *(2008) [114]	Emergency caesarean section and hysterectomy	1	-	-	-	Adverse events not reported
Schöchl *et al. *(2010) [61]	Massive trauma, then laparotomy	1	-	0	-	
Schöchl *et al. *(2010) [62]	Trauma surgery	1	-	-	-	Adverse events not reported
Schöchl *et al. *(2010) [73]	Trauma surgery	128	-	-	-	Adverse events not reported
Solomon *et al. *(2010) [72]	CV surgery	39	-	0	-	
Thorarinsdottir *et al.* (2010) [74]	Surgery (various)	37	-	0	-	
Weinkove *et al. *(2008) [115]	Various	30	-	4 (13%)	-	4 arterial ischaemic events (median 7.5 days (range 4 to 12 days) following fibrinogen concentrate administration)
Totals (noncomparator trials and case reports reporting adverse events)		240	-	7 (3%)	-	

CV = cardiovascular; RCT = randomised, controlled trial; - = no data. *Predefined safety outcomes: thrombotic events, acute lung injury, transfusion-associated circulatory overload, infections (bacterial contamination and viral transmission) and multiple organ failure. ^^^excludes deaths.

This review presents a consistent picture of the efficacy of fibrinogen concentrate and supports the safety of the product, with a low incidence of thrombotic events reported in the included studies. However, this evidence was derived from a small number of studies with a low number of patients in each arm. Whilst published studies support the efficacy and safety of fibrinogen concentrate during surgical procedures, more trials reporting outcomes from a greater number of patients are needed to reinforce these findings.

### Fresh frozen plasma and fibrinogen concentrate during surgery and massive trauma

The use of haemostatic products often occurs in response to a 'trigger', and for FFP, historically, this was based on the percentage of blood volume lost. However, there are many difficulties inherent in estimating the volume of blood lost in a patient with a life-threatening massive haemorrhage, for whom resuscitation efforts may continue for some time. Though primarily used during massive trauma bleeding, a ratio-based approach, often integrated into a massive transfusion protocol which aims to coordinate the activities of the various necessary departments and personnel, is steadily replacing the volume of blood loss as the trigger for FFP administration. This approach appears simple and practical, though concerns remain, not the least of which is that the optimum FFP:RBC ratio has yet to be determined [126], an observation highlighted in recent evidence-based guidelines [127]. In addition to uncertainty over the optimum FFP:RBC ratio, there are a number of logistical considerations, such as potential issues with the availability of thawed FFP, the waste that could be created by immediately thawing large quantities of FFP (because of the short shelf-life of thawed FFP) and the need for ABO-compatible plasma early on. Interestingly, three studies prompt questions regarding the survival benefits reported for increased FFP:RBC ratios in massive trauma patients. Snyder and colleagues [89] suggested that the reduction in mortality reported in many studies of this type is due to survival bias brought about by the exclusion from the analyses of patients who died during the early phase of the study. To test this hypothesis, they performed an analysis adjusted for survival bias and found that an FFP:RBC ratio ≥ 1:2 no longer had any effect on in-hospital mortality rates compared with < 1:2. Furthermore, both Riskin and colleagues [85] and Gunter and colleagues [28] found a significant improvement in survival after implementation of a massive transfusion protocol despite unchanged FFP:RBC ratios. This suggests that simply increasing the amount of FFP administered during a massive transfusion is not the key factor in improving survival and that other factors inherent in a transfusion protocol also have a substantial impact. The survival benefit of FFP may be due more to the focus on 'haemostasis' and the timely delivery of blood products rather than to the increase in FFP, which was reinforced in a recent review of the impact of the introduction of goal-directed haemostatic resuscitation on surgical and trauma patients [128].

When a ratio-based approach is not considered appropriate, such as during routine surgical procedures, a laboratory test-based approach is often employed to guide the administration of FFP on the basis of the prothrombin time and International Normalised Ratio. However, only the initiation phase of haemostasis is monitored by conventional coagulation tests. Screening may not indicate abnormalities, such as the presence of critically low fibrinogen levels, if the coagulopathy occurs in the amplification, propagation or stabilisation phase. The change in plasma fibrinogen levels reported in relevant studies in response to different doses of FFP and fibrinogen concentrate indicate that a good response was achieved in all studies in which fibrinogen concentrate was used, with 2 to 4 g typically raising plasma fibrinogen levels by around 1 g/L [26,54,55,59,71,72,104,113,115]. The increases in levels were generally more variable and less predictable for FFP [22,23,30,38,41,49,53,64,108-110]. According to recent measurements, because 1 L of FFP provides an average of 2.0 g of fibrinogen [16] and the same amount of fibrinogen can be found in just 100 ml of fibrinogen concentrate [17], the use of fibrinogen concentrate over FFP for substitution of fibrinogen may be more favourable. This reduced infusion volume may help avoid dilutional coagulopathy and the risk of volume overload associated with FFP use in patients with nonmassive bleeding. Conversely, in situations where a patient has experienced massive blood loss, the greater infusion volume of FFP may better restore some of the lost volume (though FFP should never be used solely as a volume expander [9,119]).

A goal-directed approach using low fibrinogen levels as a trigger for intervention could improve outcomes. The efficacy of fibrinogen concentrate to improve haemostasis has been demonstrated in a number of *in vitro *and animal models of haemodilution and severe bleeding [120,122-125,129,130]. These efficacious findings in preclinical studies are supported by the safety record of fibrinogen concentrate, such as the low thrombogenic potential demonstrated in animal models [120,124,125], and that reported in published trials. In our experience, fibrinogen concentrate has some clear advantages over FFP. The preparation method carries a reduced risk of immunological side effects, reduced risk of viral transmission, reduced transfusion volume and a known factor content [20]. In addition to the low risk of the product, its low volume facilitates faster infusion times. Furthermore, rapid administration is possible because there is no need to blood-type, thaw or warm fibrinogen concentrate.

## Conclusions

The evidence for the efficacy of FFP in our review was inconsistent across all assessed outcomes. The weight of the evidence does not appear to support the clinical effectiveness of FFP in many situations, and even suggests that it can be detrimental. The evidence was typically not of high quality, and, combined with nonstandard reporting of outcomes, drawing definitive conclusions about the benefits of FFP in the perioperative and/or massive trauma setting is difficult. In contrast, despite a low number of studies reporting outcomes associated with the perioperative administration of fibrinogen concentrate, there was a much more consistent message showing a benefit of fibrinogen concentrate over both FFP and crystalloid and colloid on a number of outcome measures, including reduction of blood loss and allogeneic transfusions. Perioperatively, the use of fibrinogen concentrate in an early goal-directed coagulation management strategy may be preferable to FFP in terms of clinical effectiveness. However, there is currently insufficient evidence in the literature to draw any definitive conclusions. More high-quality prospective studies, particularly studies directly comparing fibrinogen concentrate with FFP, are required.

## Key messages

◆ There is a relative paucity of high-quality prospective studies reporting the outcome of FFP administration in a perioperative or massive trauma setting, despite having been in use for many years.

◆ There is inconsistent and contradictory evidence concerning the efficacy of FFP. No single outcome has shown a benefit of FFP in > 50% of extracted measures.

◆ The use of fibrinogen concentrate as a haemostatic intervention in the management of perioperative bleeding is still in its early years, which is reflected by the low number of published studies that we identified.

◆ Evidence for the efficacy of fibrinogen concentrate was consistent, with no negative outcome reported for any measure.

◆ There appears to be some promise in the use of fibrinogen concentrate instead of FFP for the treatment of acquired bleeding, although there is insufficient evidence currently available in the literature to draw any definitive conclusions. More high-quality prospective studies are needed, particularly studies directly comparing fibrinogen concentrate with FFP.

## Abbreviations

ALI: acute lung injury; CTD: chest tube drainage; CV: cardiovascular surgery; FFP: fresh frozen plasma; HES: hydroxyethyl starch; LOS: length of stay; MOF: multiple organ failure; PC: platelet concentrate; RBC: packed red blood cells; RCT: randomised, controlled trial; SD-FFP: solvent/detergent-treated fresh frozen plasma; TACO: transfusion-associated circulatory overload; TRALI: transfusion-associated acute lung injury.

## Competing interests

SKL has received travel reimbursement and speaker's fees from Biotest, Octapharma, Baxter, TEM Innovations and CSL Behring; travel reimbursement and honoraria for consulting on a Biotest advisory board; and an unrestricted educational grant for the e-learning, 'perioperative bleeding', from CSL Behring. BS has participated on advisory boards and/or received speaker's honoraria from Novo Nordisk, Baxter, CSL Behring, Bayer, Pentapharm and Swedish Orphan Biovitrum GmbH. The Haemostasis Research Unit receives unrestricted research support from Novo Nordisk, Grifols, CSL Behring, LFB, Baxter, Bayer and Octapharma. JRH received travel reimbursement and an honorarium for consulting with CSL Behring.

DRS's academic department receives grant support from CSL Behring and Vifor SA (no grant numbers are attributed). DRS was chairman of the ABC Faculty and a member of the ABC Trauma Faculty, which is managed by Thomson Physicians World GmbH and sponsored by an unrestricted educational grant from Novo Nordisk A/S. DRS has received travel reimbursement and/or honoraria for consulting or lecturing from Abbott AG, AstraZeneca AG, Bayer (Schweiz) AG, Baxter SpA, B Braun Melsungen AG, Boehringer Ingelheim (Schweiz) GmbH, Bristol-Myers Squibb, CSL Behring GmbH, Curacyte AG, Ethicon Biosurgery, Fresenius SE, Galenica AG (including Vifor SA), GlaxoSmithKline GmbH & Co KG, Janssen-Cilag AG, Novo Nordisk A/S, Octapharma AG, Organon AG, Oxygen Biotherapeutics, Pentapharm GmbH (now Tem International GmbH), Roche Pharma (Schweiz) AG and Schering-Plough International.

## Authors' contributions

SKL contributed to the conception and design of the study, the acquisition of funding, the systematic literature search and the drafting of the manuscript. BS contributed to the conception of the study, the interpretation of data and the drafting of the manuscript. JH and DS contributed substantially to the interpretation of data and critical revision of the manuscript. All authors read and approved the final manuscript for publication.
